# Seasonal Incidence of Human Metapneumovirus in High‐Risk Adults With Medically Attended Acute Respiratory Illness in a Rural US Community

**DOI:** 10.1111/irv.70119

**Published:** 2025-07-17

**Authors:** Maria E. Sundaram, David L. McClure, Oluwakemi D. Alonge, Jennifer P. King, Jennifer K. Meece, Huong Q. Nguyen

**Affiliations:** ^1^ Center for Clinical Epidemiology and Population Health Marshfield Clinic Research Institute Marshfield Wisconsin USA; ^2^ Integrated Research and Development Laboratory Marshfield Clinic Research Institute Marshfield Wisconsin USA

**Keywords:** disease burden, high‐risk adults, human metapneumovirus, respiratory virus

## Abstract

**Background:**

The burden of human metapneumovirus (hMPV) among community‐dwelling high‐risk adults is understudied. We calculate the cumulative incidence of outpatient hMPV in high‐risk adults, over five consecutive winter respiratory virus seasons (2015–2016 through 2019–2020), and describe clinical characteristics of their illnesses.

**Methods:**

We conducted a retrospective analysis of data and respiratory specimens from adults ≥ 18 years old originally participating in a test‐negative study of influenza vaccine effectiveness. We included adults with ≥ 1 high‐risk condition in 2015–2016 through 2019–2020 seasons. Residual respiratory specimens were retested for hMPV using a multiplex viral panel. We calculated seasonal incidence using Poisson regression and population weighting, with the sum of observed and extrapolated hMPV cases in the study cohort divided by the number of adults with high‐risk conditions in the underlying source population.

**Results:**

We tested 3601 respiratory samples; the mean (SD) age of individuals contributing samples was 53 (19) years. We identified 289 individuals (8.0%) with a respiratory sample positive for human metapneumovirus. The estimated seasonal incidence of outpatient hMPV‐associated acute respiratory illness was 95.6 (95% CI: 80.5–113.4) cases per 10,000 high‐risk adults. These values varied by season, with the highest incidence in 2015–2016 (276.8 cases per 10,000; 95% CI: 210.7–363.5) and lowest in 2016–17 (55.0 cases per 10,000; 95% CI: 31.2–97.0).

**Conclusions:**

We identified substantial seasonal incidence of hMPV cases in community‐dwelling high‐risk adults in a Wisconsin population cohort.

**Abbreviations**
ARI: acute respiratory infection
CHF: congestive heart failure
CI: confidence interval
COPD: chronic obstructive pulmonary disease
COVID‐19: coronavirus disease‐2019
ED: emergency department
HARTI: the Hospitalized Acute Respiratory Tract Infection study
hMPV: human metapneumovirus
ICD: International Classification of Diseases
IQR: interquartile range
IRB: institutional review board
KINRESS: Korean Influenza and Respiratory Surveillance System
MCHS: Marshfield Clinic Health System
MESA: Marshfield Epidemiologic Study Area
RSV: respiratory syncytial virus
SARS‐CoV‐2: severe acute respiratory syndrome coronavirus‐2
SD: standard deviation
WSLH: Wisconsin State Laboratory of Hygiene

## 1 Background

Infection with seasonal winter respiratory viruses is known to cause a substantial burden of acute respiratory illness (ARI) among “high‐risk” adults (adults with underlying conditions that could increase the risk of a severe outcome, such as hospitalization, after infection) [1, 2, 3]. Influenza, respiratory syncytial virus (RSV), and SARS‐CoV‐2 are recognized to cause a high proportion of these illnesses; but several other respiratory viruses, including human metapneumovirus (hMPV), have also been observed to cause severe disease and hospitalization in high‐risk adults, including those who are immunocompromised, and/or who have conditions such as asthma, chronic obstructive pulmonary disease (COPD), cardiovascular disease, and diabetes [4, 5, 6, 7].

hMPV was first identified in 2001 [8] and has been implicated in wheezing and bronchitis among adults with COPD [4, 5], congestive heart failure (CHF) [5], and chronic heart disease, chronic renal disease, and asthma [4]. Though some studies have followed community cohorts of individuals to assess hMPV [4], the majority of existing research on hMPV has focused primarily on hospitalized patients [4, 6, 7, 9, 10]. Assessments of the population‐level burden of hMPV‐associated outpatient ARI in high‐risk adults have not yet been conducted.

We originally used data from this study to investigate the seasonal incidence of RSV among high‐risk adults with ARI in the outpatient setting. In this analysis, we estimate the cumulative incidence of outpatient hMPV‐associated ARI, over five consecutive winter respiratory virus seasons (2015–2016 through 2019–2020).

## 2 Methods

### 2.1 Source Population

This analysis includes individuals who participated in an influenza vaccine effectiveness study from the 2015–2016 through 2019–2020 winter respiratory virus seasons in Marshfield, Wisconsin and the surrounding geographic area. The details of these vaccine effectiveness studies have been published elsewhere [11, 12, 13, 14]. Briefly, a source population of potentially eligible individuals was established before the beginning of each winter respiratory virus season. The source population included residents of the Marshfield Epidemiologic Study Area (MESA) and individuals seen at Marshfield Clinic Health System (MCHS) medical facilities in four rural Wisconsin communities. Individuals who were not MESA residents were required to have at least two encounters in the past 3 years within MCHS, on two different dates, with one of the encounters being with a provider type of doctor of medicine, doctor of osteopathy, nurse practitioner, physician assistant, or resident at a recruitment facility. We implemented these criteria to ensure a reasonable likelihood of capturing a medically attended ARI within MCHS, for individuals deciding to seek care (i.e., to ensure that cases of ARI were not missed due to individuals seeking care outside MCHS). Dates of active study enrollment are listed in Table S1.

Individuals belonging to the source population who sought care at outpatient MCHS medical facilities for a respiratory illness with cough (i.e., a “medically‐attended acute respiratory illness”) were approached by dedicated study staff to assess eligibility for the vaccine effectiveness study. Individuals were eligible to participate if they were ≥ 6 months old, had symptom duration 7 days or less, and had not taken an influenza antiviral medication in the past 7 days. Individuals could be enrolled more than once per winter season if their previous enrollment occurred at least 14 days prior.

### 2.2 Data and Specimens Source

At the time of enrollment in the vaccine effectiveness study, participants completed an enrollment questionnaire, and authorized study team access to electronic health records to collect additional relevant clinical and demographic information. Participants provided information about their age at time of enrollment (categorized as 18–49, 50–59, 60–74, and ≥ 75 years of age); sex; race/ethnicity (Black or African‐American, Asian, Native Hawaiian or other Pacific Islander, American Indian or Alaska Native, other (including multiple races), White, and/or Hispanic); education level (less than high school graduate, high school graduate or equivalent certification, some college, bachelor's degree, or advanced degree); self‐reported general health before their illness (a 5‐point Likert scale, where 1, *Excellent*; 2, *Very good*; 3, *Good*; 4, *Fair*; and 5, *Poor*); symptom onset date; and symptoms and signs at time of enrollment (fever/feverishness, fatigue/feeling run‐down, nasal congestion, wheezing, shortness of breath, sore throat, muscle pain/myalgia, headache, and/or vomiting). For this study, we additionally extracted information from the electronic health record, including the presence of high‐risk conditions (as defined above), and Charlson Comorbidity Index score at time of enrollment [15]. In this analysis, Charlson score was used as one tool to identify potential heterogeneity in risk and/or frailty among individuals with high‐risk conditions who did vs. did not have hMPV.

At the time of enrollment into the vaccine effectiveness study, participants provided a respiratory specimen (combined nasal and throat swabs) for influenza testing. Residual respiratory specimens were archived after initial testing for influenza. For the current analysis, archived specimens were retested for the presence of hMPV via a multiplex assay, the GenMark ePlex Respiratory Pathogen Panel (RPP) [16], according to manufacturer specifications and recommendations, with appropriate quality controls. The panel identifies hMPV among a panel of several viral and bacterial targets [16]. Individuals testing negative for hMPV may have tested positive for other, non‐hMPV pathogens, or may have tested negative for all targets in the multiplex panel.

### 2.3 Analytic Sample

Participants of these vaccine effectiveness studies were included in this analysis if they were ≥ 18 years old and had at least 1 underlying health condition that was considered to increase their risk of severe illness during an episode of ARI (“high‐risk condition”). High‐risk conditions were defined by having at least one applicable International Classification of Diseases (ICD) code documented in the participant's electronic health record during specific time frames relative to an index date of September 1 preceding the start of the winter respiratory season of enrollment and identified from the past 6 months to ever, depending on the condition (Table S2). High‐risk conditions were grouped into the following categories: cardiac disorders (arrhythmias, heart failure, and coronary artery disease), chronic respiratory diseases (COPD, asthma, and cystic fibrosis), chronic liver disease, chronic kidney disease, diabetes, and immunocompromised status (the presence of transplant, the presence of malignancy, current treatment with immunosuppressive medication, and other immunosuppressive disorders). Malignancy was a category including the following: lymphomas (ICD‐10 codes C81*‐C86*, C88*), multiple myeloma and malignant plasma cell neoplasms (C90*), leukemias (C91*‐C95*), and other and unspecified malignant neoplasms of lymphoid, hematopoietic, and related tissue (C96*). Further information about categorization of ICD codes can be found in Table S3.

### 2.4 Statistical Methods

We estimated the seasonal incidence of medically attended hMPV cases per 10,000 adults with high‐risk conditions, using stratification and weighting methods similar to those used in previous estimates of RSV incidence [12, 13] and influenza incidence [12]. Specifically, we used data on adults with high‐risk conditions within the vaccine effectiveness study's enrolled population and the source population. The populations of adults with high‐risk conditions in the vaccine effectiveness study and source population were stratified into mutually exclusive groups based on age category *a* (18–49, 50–59, 60–74, and ≥ 75 years), an individual's number of medically attended ARI visits *m* during a given study enrollment period, sex *g*, MESA residency status *r*, and season *s*.

A sampling weight was calculated for each high‐risk adult enrollee in the vaccine effectiveness study, per (*a, m, g, r, s*) stratum. This sampling weight was equivalent to the ratio of the number of adults with high‐risk conditions in the source population in a given (*a, m, g, r, s*) stratum, to the number of adults with high‐risk conditions in the enrolled study population in that stratum. We used these sampling weights to estimate the total number of medically attended hMPV infections in each stratum among adults with high‐risk conditions in the source population.

We also used hMPV surveillance data provided by the Wisconsin State Laboratory of Hygiene (WSLH) to further weight our incidence estimates for each respiratory virus season represented in study data [17]. This was necessary to account for the shorter enrollment intervals, geared towards identifying influenza, compared to longer seasonal intervals with later peaks associated with hMPV [18, 19, 20]. For each season, we multiplied the sampling weights by a ratio defined as the total number of WSLH hMPV‐positive counts, divided by the sum of WSLH hMPV‐positive counts, occurring during the shorter enrollment interval. Adjustment factors by season are presented in Table S4.

We used Poisson regression with analytic weights, offsets, and robust variance estimation [21, 22] to estimate seasonal incidence with corresponding 95% confidence intervals (CI) and to perform statistical tests comparing subgroups by winter respiratory virus season, age, and sex. We calculated the numerator as the number of hMPV cases in the vaccine effectiveness study population and then estimated the number of cases in the source population. Offset terms were stratified by winter respiratory virus season, age group, number of medically attended ARI visits, and sex. Acknowledging that high‐risk condition categories may vary substantially by age, cumulative incidence estimates were also calculated by high‐risk condition within age categories. Each offset was the natural log of the number of cohort members in a stratum divided by the total sum of analytic (numerator) weights. Incidence estimations were based on two key assumptions: First, test results from enrolled patients with medically attended ARI could be extrapolated to the nonenrolled source population, based on the number of medically attended ARI visits per season; and second, hMPV cases occurring outside the enrollment period for the vaccine effectiveness study were proportional to hMPV cases identified by the WSLH during that time period.

### 2.5 Ethics

The MCHS Institutional Review Board (IRB) reviewed and approved this study with a waiver of informed consent.

## 3 Results

**FIGURE 1:**
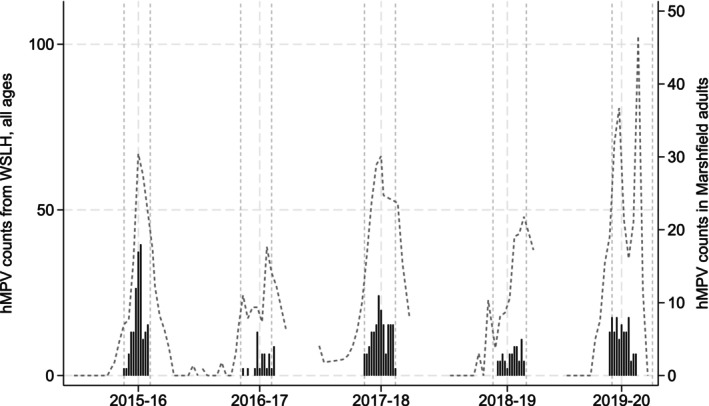
Medically‐attended hMPV cases in high‐risk Marshfield study participants ≥18 years old (black bars) and statewide hMPV counts (all ages) based on testing at the Wisconsin State Laboratory of Hygiene (dark gray dashed line).

There were a total of 3604 enrollments among high‐risk adults from 2015 to 2016 through 2019 to 2020, all of which were associated with a collected respiratory sample; of these, three samples (< 0.01%) were not able to be tested due to low sample quality. This resulted in 3601 enrollments included in the analysis from 2015 to 2016 through 2019 to 2020 seasons [a total of 89 (2.5%) individuals had more than one enrollment in the same season], of which 289 enrollments (8.0%) were associated with a respiratory sample positive for hMPV (Table 1, Figure 1). There was one individual who was enrolled in two separate seasons who tested positive for hMPV in both seasons. Nearly half of enrollments occurred in individuals ≥ 60 years of age (49.8%); a majority of participants were female (67.1%) and White (97.9%).

**TABLE 1:** Demographic characteristics and specific high‐risk conditions among adults with and without human metapneumovirus.^a^

	hMPV‐negative *n* = 3312		hMPV‐positive *n* = 289		*p* ^b^
Season: *n* (%)					< 0.01
2015–2016	409	12.3	82	28.4	
2016–2017	670	20.2	25	8.7	
2017–2018	786	23.7	81	28.0	
2018–2019	689	20.8	33	11.4	
2019–2020	758	22.9	68	23.5	
Age in years: *n* (%)					< 0.01
18–49	1372	41.4	85	29.4	
50–59	637	19.2	60	20.8	
60–74	832	25.1	94	32.5	
≥ 75	471	14.2	50	17.3	
Female sex: *n* (%)	2164	65.3	194	67.1	0.54
Race: *n* (%)^c^					0.79
Black or African‐American	18	0.5	< 5	—	
Asian	20	0.6	< 5	—	
Native Hawaiian or other Pacific Islander	< 5	—	< 5	—	
American Indian or Alaska Native	7	0.2	< 5	—	
Other (includes multiple races)	57	1.7	< 5	—	
White	3204	96.7	283	97.9	
Education: *n* (%)^d^					0.15
Less than high school	260	7.9	14	4.8	
High school graduate or obtained GED	1160	35.0	100	34.6	
Some college	1293	39.0	118	40.8	
Bachelor's	373	11.3	39	13.5	
Advanced degree	220	6.6	16	5.5	
Self‐rated general health before illness: median (IQR)^e^	2	1.0	2	1.0	0.33
Charlson score: mean (SD)	1.1	0.03	1.4	0.09	0.02
Charlson score: median (IQR)	1	0–2	1	0–2	< 0.01
High‐risk conditions					
Any non‐immunocompromising condition	3288	99.3	287	99.3	0.95
Cardiac disorders	2267	68.4	203	70.2	0.53
Congestive heart failure	297	9.0	25	8.7	0.86
Arrhythmias	2111	63.7	185	64.0	0.93
Coronary artery disease	570	17.2	56	19.4	0.35
Chronic respiratory disorders	1465	44.2	103	35.6	< 0.01
Asthma	1043	31.5	75	26.0	0.05
COPD	599	18.1	42	14.5	0.13
Cystic fibrosis	< 5	—	< 5	—	—
Chronic liver disease	449	13.6	45	15.6	0.34
Chronic kidney disease	1020	30.8	89	30.8	1.00
Diabetes	818	24.7	90	31.1	0.02
Any immunocompromising condition	202	6.1	24	8.3	0.14
Transplant	93	2.8	9	3.1	0.76
Malignancy	129	3.9	17	5.9	0.10
Immunosuppressive medications received in the past 6 months	11	0.3	< 5	—	1.00
Other immunodeficiencies	68	2.1	5	1.7	0.71
Number of high‐risk conditions in separate categories					0.51
1	1583	47.8	133	46.0	
2	917	27.7	76	26.3	
3	518	15.6	55	19.0	
4	294	8.9	25	8.7	

^a^ Where cell values are less than 5, cells have been replaced with “< 5” and percentage estimates have been replaced with “—” to preserve patient privacy.

^b^ *p*‐Values provided are the result of chi‐squared tests (for categorical variables) or Fisher's exact tests (for categorical variables where any cell in a two‐by‐two table comparison had a value < 5); Wilcoxon rank‐sum tests (for continuous variables where median [IQR] is reported); or *t*‐tests (for continuous variables where mean [SD] is reported).

^c^ Four individuals declined to provide information about their race and ethnicity.

^d^ Eight individuals declined to provide information about their education.

^e^ Self‐rated general health before illness was rated on a 5‐point Likert scale, with 1 = poor, 2 = fair, 3 = good, 4 = very good, and 5 = excellent.

The corresponding underlying source population from 2015 to 2016 through 2019 to 2020 consisted of 251,413 high‐risk adults. Compared to the enrolled cohort, the source population had a lower proportion of individuals who were 18–49 years and higher proportion of individuals ≥ 75 years old, and a slightly lower proportion of females compared to males (Table S4).

Among the enrolled population, self‐rated health before illness and Charlson comorbidity scores were similar between enrollments with and without hMPV, though Charlson score was slightly higher in individuals with hMPV. There were a higher proportion of individuals aged 60–74 and ≥ 75 years with hMPV, compared to individuals without hMPV. The presence of specific high‐risk conditions was generally similar between enrollments with and without hMPV, with hMPV‐positive enrollments having a slightly higher proportion of individuals with diabetes, and slightly lower proportion of individuals with chronic respiratory disorders, compared to hMPV‐negative enrollments. Additional relevant characteristics are found in Table 1. The clinical characteristics of medically attended ARI episodes were similar among enrollments with and without hMPV, with a slightly higher proportion of hMPV‐associated enrollments developing pneumonia within 30 days after medically attended ARI compared to enrollments without hMPV. A total of 15 individuals (5.2% of total hMPV infections) were coinfected with hMPV and another pathogen (seven cases of seasonal coronavirus, one influenza A(H1N1)pdm09, two influenza A(H3N2), one influenza B, three human rhinovirus/enterovirus, and one adenovirus). Among 3312 individuals without an hMPV infection, 1344 (40.6%) tested negative for all targets in the respiratory panel (Table 2).

**TABLE 2:** Illness characteristics and other respiratory pathogens present, among enrollments negative and positive for hMPV.

	hMPV‐negative *n* = 3312		hMPV‐positive *n* = 289	
	*n*	%	*n*	%
Days from symptom onset to enrollment				
0	68	2.1	3	1.0
1	393	11.9	17	5.9
2	588	17.8	56	19.4
3	675	20.4	58	20.1
4	539	16.3	59	20.4
5	390	11.8	35	12.1
6	301	9.1	33	11.4
7	358	10.8	28	9.7
Symptoms present during medically attended ARI visit^a^				
Fever/feverishness	2143	64.7	160	55.4
Fatigue	3093	93.4	272	94.1
Nasal congestion	2720	82.1	242	83.7
Wheezing	1991	60.1	216	74.7
Shortness of breath	2182	65.9	210	72.7
Sore throat	2246	67.8	172	59.5
Muscle pain/myalgia^b^	532	70.2	48	70.6
Headache^b^	583	76.9	51	75.0
Nausea/vomiting^b^	276	36.4	25	36.8
Pneumonia within 30 days after medically attended ARI visit^c^	135	4.1	21	7.3
Hospitalization within 30 days after medically attended ARI visit	86	2.6	10	3.5
Other respiratory pathogens present^d^				
Adenovirus	25	0.8	1	0.4
Seasonal coronaviruses^e^	339	10.2	7	2.4
Influenza A(H3N2)	340	10.3	2	0.7
Influenza A(H1N1)09	414	12.5	1	0.4
Influenza B	225	6.8	1	0.4
Human rhinovirus/enterovirus	238	7.2	3	1.0
Parainfluenza virus^f^	84	2.5	0	0
RSV A	120	3.6	0	0
RSV B	184	5.6	0	0
*Chlamydia pneumoniae*	7	0.2	0	0
*Mycoplasma pneumoniae*	33	1.0	0	0
No other respiratory pathogens present	1344	40.6	274	94.8

^a^ Study participants were required to have cough in order to be eligible to participate. Therefore, cough is not reported among these symptoms.

^b^ Muscle pain/myalgia, headache, and vomiting were assessed in the 2019–2020 season only.

^c^ The presence of pneumonia defined as individuals with at least one ICD code relating to pneumonia, following symptom onset preceding a specific medically attended ARI visit (ICD‐9: 480.0, 480.1, 480.2, 480.3, 480.8, 480.9, 481, 482.0, 482.31, 482.32, 482.39, 482.40, 482.41, 482.42, 482.49, 482.81, 482.82, 482.83, 482.84, 482.89, 482.9, 483.0, 483.1, 483.8, 484.1, 484.3, 484.5, 484.6, 484.7, 484.8, 485, 486, 487.0, 487.1, 487.8; ICD‐10: A37.01, A37.11, A37.81, A37.91, B25.0, B77.81, J12.2, J12.3, J12.81, J12.89, J12.9, J13, J14, J15.0, J15.1, J15.20, J15.211, J15.212, J15.29, J15.3, J15.4, J15.5, J15.6, J15.7, J15.8, J15.9, J16.0, J16.8, J17, J18.0, J18.1, J18.8, J18.9), plus a code indicating the presence of a chest X‐ray (ICD‐10 procedure code: BW03ZZZ; CPT codes: 71010, 71,015, 71,020, 71,021, 71,022, 71,023, 71,030, 71,034, 71,035, 71,045, 71,046, 71,047, 71,048, 71,100, 71,101, 71,110, 71,111, 71,260, 71,270).

^d^ Column percentages for other pathogens present in hMPV‐negative enrollments add up to greater than 100% because individuals could be coinfected with several non‐hMPV pathogens.

^e^ “Seasonal coronaviruses” is a category that includes HKU1, OC43, NL63, and 229E.

^f^ Parainfluenza viruses 1–4 were included in this category.

The estimated seasonal incidence of outpatient hMPV‐associated ARI was 95.6 (95% CI: 80.5–113.4) cases per 10,000 high‐risk adults. These values varied by season, with the highest incidence in 2015–2016 (276.8 cases per 10,000; 95% CI: 210.7–363.5) and lowest in 2016–2017 (55.0 cases per 10,000; 95% CI: 31.2–97.0). Incidence estimates were highest for individuals 50–59 years (141.6 cases per 10,000; 95% CI: 100.7–198.9) and individuals ≥ 75 years (147.1 cases per 10,000; 95% CI: 113.4–190.7). Incidence estimates varied by high‐risk condition, with slightly higher incidence estimates for individuals with coronary artery disease (138.1 cases per 10,000; 95% CI: 95.7–199.3), diabetes (143.7 cases per 10,000; 95% CI: 106.8–193.3), and immunocompromising conditions (134.7 cases per 10,000; 95% CI: 86.2–210.7); many estimates had wide 95% confidence intervals. Additional season‐specific and condition‐specific estimates are found in Table 3.

**TABLE 3:** Cumulative incidence of human metapneumovirus per 10,000 high‐risk adults, 2015–2016 through 2019–2020.

	hMPV cases per 10,000 high‐risk adults	
	Seasonal incidence^a^, ^b^	95% CI
Overall	95.6	80.5–113.4
Season		
2015–2016	276.8	210.7–363.5
2016–2017	55.0	31.2–97.0
2017–2018	109.7	80.5–149.5
2018–2019	60.2	37.2–97.3
2019–2020	74.5	51.6–107.6
Sex		
Female	92.8	75.4–114.1
Male	102.8	76.2–138.8
Age group		
18–49 years	65.4	48.9–87.6
50–59 years	141.6	100.7–198.9
60–74 years	69.8	34.5–141.2
≥ 75 years	147.1	113.4–190.7
High‐risk conditions		
Any condition that is not immunocompromising	95.8	80.7–113.8
Cardiac disorders	97.4	79.6–119.3
Heart failure	88.8	56.2–140.4
Arrhythmias	95.0	77.0–117.3
Coronary artery disease (CAD)	138.1	95.7–199.3
Chronic respiratory conditions	78.3	60.9–100.7
Asthma	77.5	57.4–104.5
COPD	92.4	64.3–132.9
Cystic fibrosis^c^	—	—
Chronic liver conditions	115.8	75.3–178.0
Chronic kidney disorders	96.6	72.5–128.7
Diabetes	143.7	106.8–193.3
Any immunocompromising condition	134.7	86.2–210.7
Malignancy	87.8	41.0–188.2
Transplant	150.2	87.7–257.4
Immunosuppressive medications^d^	—	—
Other immunodeficiencies	83.0	30.22–227.8

Abbreviation: CI, confidence interval.

^a^ Seasonal incidence calculated using a Poisson regression with a log link, sampling weights, offsets, and robust variance estimation. Adjustment factors are additionally applied based on the estimated true length of the hMPV season, according to Wisconsin State Laboratory of Hygiene reports for each season. This adjustment factor is based on the inverse of the proportion of all statewide cases that occurred during seasonal enrollment periods. Wisconsin hMPV cases were determined for the period Week 40 through Week 18, and for the specific study enrollment window each winter using data reported by the Wisconsin State Laboratory of Hygiene. Confidence intervals are Wald 95% confidence intervals. Offset terms are stratified by winter respiratory virus season, age group, number of medically attended ARI encounters per season, and sex. Each offset is the natural log of number of individuals in a sampling stratum divided by total sum of analytic weights in the stratum.

^b^ Estimates generated by extrapolating the number of medically attended acute respiratory infections among adults in the high‐risk adult cohort, to the high‐risk adult source population.

^c^ There were no hMPV‐positive individuals with cystic fibrosis in this analysis.

^d^ There were no hMPV‐positive individuals receiving immunosuppressive medications in this analysis.

## 4 Discussion

Among high‐risk adults with outpatient medically attended ARI during a winter respiratory virus season, approximately 8% of illnesses had infection with hMPV; we estimated approximately 95.6 cases of outpatient hMPV per 10,000 high‐risk adults per respiratory virus season. These findings suggest a seasonal incidence of outpatient hMPV in high‐risk adults similar to other estimates we have produced on outpatient RSV incidence in high‐risk adults. Additionally, our findings that hMPV prevalence may differ according to age and may exhibit variability across winter respiratory virus seasons are supported by existing literature suggesting that hMPV prevalence may be higher in older adults [4, 5, 9, 23, 24] and may have substantial heterogeneity by season [5, 9].

A substantial portion of the existing research on hMPV epidemiology has focused on hMPV identified in emergency department or inpatient settings (thereby de‐emphasizing the burden of hMPV‐associated ARI in medically attended outpatient visits) and has yielded heterogeneous results. A large, prospective, multicountry study (HARTI, the Hospitalized Acute Respiratory Tract Infection study) identified 107 hMPV cases (2.8%) among 3861 adults hospitalized with acute respiratory tract infection from 2017 to 2019 [4]. However, data from the Japanese cohort of the HARTI prospective cohort study identified 11 hMPV cases (6.4%) among 173 adults hospitalized with acute respiratory tract illness from 2018 to 2019 [10]. Additional studies using administrative datasets have estimated the burden of hMPV among hospitalized individuals in US hospitals at 12.1 cases of hMPV per 100,000 adult hospitalizations per year using the National Inpatient Sample from 2016 to 2019 [23], or at 39–65 cases of hMPV per 100,000 adult hospitalizations per year using information from ARI hospitalizations in Pennsylvania from 2015 to 2019 [25].

It is challenging to directly compare estimates of hMPV prevalence from our study to estimates of hMPV in hospitalized adults. However, an analysis of inpatient and outpatient ARI in the Korean Influenza and Respiratory Surveillance System (KINRESS) estimated the prevalence of hMPV to range from 5.6%–6.9% from 2015 to 2019 [18]. Our estimates of hMPV prevalence are comparatively slightly higher, likely because our analysis only included individuals with high‐risk conditions and identified enrollments using dedicated study staff (vs. the sentinel surveillance methodology underlying the structure of KINRESS).

Our analysis identified potential, though not statistically significantly different, changes in the incidence of hMPV according to particular high‐risk conditions. Several cohort studies have investigated the burden of hMPV among individuals with specific high‐risk conditions, with varying estimates on the impact of hMPV in these populations. A study which followed a total of 537 individuals with symptomatic lung disease (largely COPD or CHF) prospectively for a maximum of two consecutive winters (study duration: 1999–2003) identified 524 ARI, of which 49 (9.3%) were hMPV infections [5]. This was slightly higher than the proportion of hMPV‐positive illnesses identified in a similar cohort of otherwise healthy elderly subjects (6.9%) [5]. Two smaller studies identified similar prevalence of hMPV among individuals with COPD exacerbation: a prospective cohort study of 86 individuals with COPD identified hMPV viruses to be implicated in seven instances of COPD exacerbation from 2007 to 2008 [26] and an observational cohort study of 50 individuals hospitalized due to COPD exacerbation identified hMPV present in six (12%) of individuals [27]. Finally, a prospective cohort study of 101 adults hospitalized for acute asthma exacerbation identified hMPV present in seven (6.9%) of subjects during hospitalization [28]. These findings mirror our COPD‐ and asthma‐specific estimates of hMPV‐associated ARI incidence.

The burden of hMPV has also been investigated specifically among individuals with underlying conditions affecting immunologic function, such as cystic fibrosis and history of organ transplant. A prospective analysis of patients with cystic fibrosis identified 0.28 cases of hMPV (95% CI: 0.17–0.39) per patient‐year, among a cohort of 100 individuals with cystic fibrosis [29]. That analysis identified 13% of viruses during the study as hMPV (only 3.6% were influenza and 2.0% were RSV). In a retrospective review of electronic health records of 23 solid organ transplant recipients with community‐acquired respiratory viruses from 2014 to 2019, hMPV was identified among 12% of the cohort [30]. Conversely, an analysis from a prospective cross‐sectional study of stem cell transplant recipients identified only 1 case of hMPV among 85 subjects with 31 respiratory viral infection specimens available for testing [31]. By comparison, our analysis identified higher overall point estimates for hMPV‐associated ARI incidence among individuals with immunocompromising conditions, and specifically for individuals with a history of transplant (though 95% confidence intervals were wide).

Our analysis is similar to our own published results on the incidence of RSV in the same population of high‐risk adults. Similarity between the burden of hMPV and RSV in the same population was also reported in a prior investigation of ARI etiology associated with ED visits and hospitalizations for respiratory symptoms or nonlocalizing fever, during May 2009–April 2010 [6]. In that analysis, hMPV infection incidence (14.6 cases per 10,000; 95% CI: 8.6–24.7) was also estimated to be similar to RSV infection incidence (15.4 cases per 10,000; 95% CI: 9.3–25.4) in adults ≥ 18 years old seeking care in the emergency department. Similar results were also observed in the abovementioned HARTI‐based study of 173 hospitalized adults in Japan [10], as well as a large‐scale analysis of hMPV and RSV cases in China [32]. However, other analyses identifying both RSV and hMPV in hospitalized patients have identified a higher burden of RSV compared to hMPV [4, 23, 33].

## 5 Limitations

This study has several limitations. First, existing literature on the seasonality of hMPV suggests that its overlap with influenza and RSV seasons is not perfect, with hMPV epidemic peaks in later winter/early spring [18, 20]. The results of this analysis are based on information collected primarily during times of influenza transmission, meaning that cases of hMPV could have been missed if they occurred after study enrollment for influenza ended. We attempted to mitigate the potential for this bias by adjusting our estimates according to WSLH information about hMPV circulation in the state. However, this adjustment may be imperfect; cases of hMPV reported to WSLH are a convenience sample and may be biased according to age, meaning that they may not provide a complete picture of population‐level hMPV circulation.

Additionally, this analysis presents descriptive comparisons between individuals who are hMPV‐positive versus hMPV‐negative. However, coinfections are included among hMPV‐positive individuals; similarly, hMPV‐negative individuals could have infection with another respiratory pathogen, including coinfection with two or more non‐hMPV respiratory pathogens, or could test negative for all targets in the respiratory panel. The presence of other respiratory pathogens in this study is an accurate reflection of real‐world community risk of respiratory illness, and the structure of the underlying study allows for assumptions that illness severity is reasonably comparable at the time of study enrollment. However, the possibility of other respiratory pathogens may obscure the assessment of potential relationships between hMPV single infection and long‐term or severe outcomes.

Finally, this study presents results from the 2015–2016 through 2019–2020 respiratory virus seasons. However, existing evidence suggests that epidemic curves and peaks of hMPV may have shifted during the COVID‐19 pandemic [32, 34, 35, 36]. Continued investigation is needed to appropriately define season start, stop, and epidemic peaks of hMPV during the post‐COVID‐19 era.

## 6 Conclusions

Our analysis identified substantial incidence of hMPV in adults 18 years and older with high‐risk conditions over the course of five winter respiratory virus seasons. Incidence estimates varied by high‐risk condition, with slightly higher incidence estimates for individuals with coronary artery disease, diabetes, and immunocompromising conditions, though 95% confidence intervals were wide. These findings support the continued surveillance and assessment of hMPV, especially in adults with high‐risk conditions.

## Author Contributions

**Maria E. Sundaram:** conceptualization, investigation, funding acquisition, writing – original draft, visualization, writing – review and editing, formal analysis, supervision. **David L. McClure:** conceptualization, methodology, visualization, formal analysis, writing – review and editing, validation. **Oluwakemi D. Alonge:** project administration, writing – review and editing, resources. **Jennifer P. King:** writing – review and editing, project administration, resources. **Jennifer K. Meece:** writing – review and editing, funding acquisition. **Huong Q. Nguyen:** conceptualization, writing – review and editing, funding acquisition, methodology, supervision.

## Conflicts of Interest

M.E.S. received funding from GSK Inc., for the completion of sample analysis and a portion of data analysis for this project. M.E.S., O.D.A., J.P.K. and D.L.M. received research support from GSK Inc., for the primary study on which this analysis is based. ODA receives additional research support from CSL Seqirus and ModernaTX. H.Q.N. receives research support from CSL Seqirus, ModernaTX, and GSK, and an honorarium for participating in a consultancy group for ModernaTX. Other authors declare no conflicts of interest.

## Supporting information


**Table S1.** Enrollment period for the vaccine effectiveness study, by season.^a^

**Table S2.** ICD‐10 codes used to identify an individual as having a high‐risk condition.

**Table S3.** ICD codes used for definitions of specific high‐risk subcategories among individuals in the high‐risk adult cohort.

**Table S4.** Summary statistics for population‐weighting factors in the high‐risk adult cohort and high‐risk adult source population.

## Data Availability

The data used in this analysis are not publicly available.
