# Supplementary material for: Seasonal Incidence of Human Metapneumovirus in High‐Risk Adults With Medically Attended Acute Respiratory Illness in a Rural US Community
Source: Influenza Other Respir Viruses. 2025 Jul 17;19(7):e70119. doi: 10.1111/irv.70119 (PMC12268109; doi:10.1111/irv.70119)
Supplement: Supplementary file 1 — Table S1. Enrollment period for the vaccine effectiveness study, by season.a [file IRV-19-e70119-s001.docx]

**Supplemental Table 1**. Enrollment period for the vaccine effectiveness study, by season.^a^

| Season | Season start and end dates |
| --- | --- |
| 2015-16 | 18 Jan 2016 – 8 Apr 2016 |
| 2016-17 | 3 Jan 2017 – 14 Apr 2017 |
| 2017-18 | 26 Dec 2017 – 6 Apr 2018 |
| 2018-19 | 14 Jan 2019 – 26 Apr 2019 |
| 2019-20 | 30 Dec 2019 – 14 Mar 2020 |

# ^a^ Local enrollment was triggered by 2 consecutive weeks of increasing detection of influenza viruses by real-time RT-PCR assays conducted as part of pre-enrollment surveillance. Increasing detection was based on week-to-week increase in the total number of positives and the proportion positive among those tested. Enrollment continued for at least 10 weeks; the end of the enrollment period was defined by at least 1 week with no influenza detections among subjects enrolled in the study, or as long as possible based on funding, if influenza activity was ongoing.
