# Supplementary material for: Seasonal Incidence of Human Metapneumovirus in High‐Risk Adults With Medically Attended Acute Respiratory Illness in a Rural US Community
Source: Influenza Other Respir Viruses. 2025 Jul 17;19(7):e70119. doi: 10.1111/irv.70119 (PMC12268109; doi:10.1111/irv.70119)
Supplement: Supplementary file 2 — Table S2. ICD‐10 codes used to identify an individual as having a high‐risk condition. [file IRV-19-e70119-s002.docx]

# **Supplemental Table 2.** ICD-10 codes used to identify an individual as having a high-risk condition.

| **Condition^a^** | **ICD-10 code** | **Time frame^b^** | **ICD-9 codes (if needed)** |
| --- | --- | --- | --- |
| **Chronic respiratory diseases** |  |  |  |
| Aspergillosis | B44* | Past 5 years | 117.3 |
| Cystic fibrosis | E84* | Ever | 277.01, 277.02, 277.03 |
| Simple and mucopurulent chronic bronchitis | J41 (J41.0, J41.1, J41.8) | Ever | 491.0, 491.1, 491.8 |
| Unspecified chronic bronchitis | J42* | Ever | 491.9 |
| Emphysema | J43* | Ever | 492.0, 492.8 |
| MacLeod syndrome | J43.0 | Ever | 492.8 |
| Panlobular emphysema | J43.1 | Ever | 492.8 |
| Centrilobular emphysema | J43.2 | Ever | 492.8 |
| Other emphysema | J43.8 | Ever | 492.8 |
| Emphysema, unspecified | J43.9 | Ever | 492.0, 492.8 |
| Other chronic obstructive pulmonary disease | J44 (J44.0, J44.1, J44.9) | Ever | 491.22, 493.21, 491.21, 493.22, 491.20, 493.20, 496 |
| Asthma | J45* | Past 5 years | 493.00 – 493.99 |
| Bronchiectasis | J47* | Ever | 494.0, 494.1 |
| Coalworker’s pneumoconiosis | J60* | Ever | 500 |
| Pneumoconiosis due to asbestos and other mineral fibers | J61* | Ever | 501 |
| Pneumoconiosis due to dust containing silica | J62* | Ever | 502 |
| Aluminosis (of lung), bauxite fibrosis (of lung), berylliosis, graphite fibrosis (of lung), siderosis, and stannosis | J63.0 to 63.5 | Ever | 503 |
| Farmer’s lung, bagassosis, bird fancier’s lung, suberosis, maltworker’s lung, mushroom-worker’s lung, maple-bark-stripper’s lung, air conditioner and humidifier lung, and hypersensitivity pneumonitis due to other organic dusts | J67.0 to 67.8 | Ever | 495.0, 495.1, 495.2, 495.3, 495.4, 495.5, 495.6, 495.7, 495.8 |
| Chronic respiratory conditions due to chemicals, gases, fumes and vapours | J68.4 | Ever | 506.4 |
| Acute respiratory distress syndrome | J80* | Past 5 years | 518.82 |
| Pulmonary eosinophilia, not elsewhere classified | J82* | Ever | 518.3 |
| Other interstitial pulmonary diseases | J84* | Ever | 515, 516.0, 516.1, 516.30, 516.31, 516.32, 516.33, 516.34, 516.37, 516.8, 516.9 |
| Chronic respiratory failure | J96.1* | Ever | 518.83 |
| Interstitial emphysema | J98.2* | Ever | 518.1 |
| Respiratory disorders in diseases classified elsewhere | J99* | Ever | 517.8 |
|  |  |  |  |
| **Chronic liver disease** |  |  |  |
| Chronic viral hepatitis | B18* | Ever | 070.32, 070.33, 070.54, 070.59 |
| Glycogen storage disease | E74.0* | Ever | 271.0 |
| Disorders of copper metabolism | E83.0* | Ever | 275.1 |
| Disorders of iron metabolism | E83.1* | Ever | 275.01, 275.02, 275.03, 275.09 |
| Alcoholic liver disease (alcoholic fatty liver, alcoholic hepatitis, alcoholic fibrosis and sclerosis of liver, alcoholic cirrhosis of liver) | K70.0 to K70.3 | Ever | 571.0, 571.1, 571.2 |
| Alcoholic hepatic failure | K70.4*, K70.40, K70.41 | Ever | 571.3 |
| Alcoholic liver disease, unspecified | K70.9 | Ever | 571.3 |
| Toxic liver disease | K71 (K71.0, K71.1, K71.10, K71.11, K71.2, K71.3, K71.4, K71.5, K71.50, K71.51, K71.6, K71.7, K71.8, K71.9) | Ever | 573.3 |
| Hepatic failure, not elsewhere classified | K72 (K72.0, K72.00, K72.01, K72.10, K72.11, K72.9, K72.90, K72.91) | Ever | 570, 572.2, 572.8 |
| Chronic hepatitis, not elsewhere classified | K73* | Ever | 571.40, 571.41, 571.49 |
| Fibrosis and cirrhosis of liver | K74.0* to K74.6* | Ever | 571.5, 571.6, 571.9 |
| Nonspecific reactive hepatitis | K75.2 | Ever | 573.3 |
| Granulomatous hepatitis, not elsewhere classified | K75.3 | Ever | 573.3 |
| Auto-immune hepatitis | K75.4 | Ever | 571.42 |
| Other specified inflammatory liver diseases | K75.8* | Ever | 571.8, 573.3 |
| Fatty (change of) liver, not elsewhere classified | K76.0 | Ever | 571.8 |
| Hepatorenal syndrome | K76.7 | Ever | 572.4 |
|  |  |  |  |
| **Cardiac disorders** |  |  |  |
| Rheumatic heart failure | I09.81 | Ever | 398.91 |
| Hypertensive heart disease with heart failure | I11.0 | Ever | 402.01, 402.11, 402.91 |
| Angina pectoris | I20* | Between 5 years before and 6 months before index date^a^ | 413.0, 413.1, 413.9 |
| Acute myocardial infarction | I21* | Between 5 years before and 6 months before index date^a^ | 410* |
| Subsequent ST elevation (STEMI) and non-ST elevation (NSTEMI) myocardial infarction | I22* | Between 5 years before and 6 months before index date^a^ | 410* |
| Certain current complications following ST elevation (STEMI) and non-ST elevation (NSTEMI) myocardial infarction (within the 28 day period) | I23* | Past 5 years | 429.79, 429.71, 429.5, 429.6 |
| Hypertensive heart and chronic kidney disease | I13* | Ever | 404.00, 404.01, 404.02, 404.03, 404.10, 404.11, 404.12, 404.13, 404.90, 404.91, 404.92, 404.93 |
| Other acute ischemic heart diseases | I24* | Between 5 years before and 6 months before index date^a^ | 411.81, 411.0, 411.89 |
| Chronic ischemic heart disease | I25* | Ever | 414.01, 414.02, 414.03, 414.04, 414.05, 414.06, 414.07, 414.10, 414.11, 414.12, 414.19, 414.2, 414.3, 414.4, 414.8, 414.9, 429.2, 412 |
| Other pulmonary heart diseases | I27* | Ever | 416.0, 416.1, 416.2, 416.8, 416.9 |
| Cardiomyopathy | I42* | Ever | 425.0, 425.11, 425.18, 425.3, 425.4, 425.5, 425.9 |
| Atrioventricular and left bundle-branch block | I44 (I44.0, I44.1, I44.2, I44.3, I44.30, I44.39, I44.4, I44.5, I44.6, I44.60, I44.69, I44.7) | Ever | 426.11, 426.12, 426.13, 426.0, 426.10, 426.50, 426.2, 426.3 |
| Other conduction disorders | I45* | Ever | 426.4, 426.50, 426.51, 426.52, 426.53, 426.54, 426.6, 426.7, 426.81, 426.82, 426.89, 426.9 |
| Atrial fibrillation and flutter | I48* | Ever | 427.31, 427.32 |
| Other cardiac arrhythmias | I49.1, I49.2, I49.3, I49.4, (I49.4, I49.40, I49.49), I49.5, I49.8, I49.9 | Ever | 427.41, 427.42, 427.61, 427.0, 427.69, 427.60, 427.69, 427.81, 427.89, 427.9 |
| Encounter for adjustment and management of implanted device | Z45.0, Z45.01, Z45.010, Z45.018, Z45.02, Z45.09 | Past 1 year | V53.31, V53.32, V53.39 |
| Heart failure | I50* | Ever | 428.0, 428.1, 428.20, 428.21, 428.22, 428.23, 428.30, 428.31, 428.32, 428.33, 428.40, 428.41, 428.42, 428.43, 428.9 |
| Presence of cardiac and vascular implants and grafts; other postprocedural states | Z95.0, Z95.1, Z95.5, Z95.810, Z98.61 | Ever | V45.01, V45.81, V45.82, V45.02 |
| Abnormalities of heart beat | R00.0, R00.1, R00.2 | Ever | 785.0, 427.81, 427.89, 785.1 |
|  |  |  |  |
| **Chronic kidney disease** |  |  |  |
| Lipoprotein deficiency | E78.6 | Ever | 272.5 |
| Non-neuropathic heredofamilial amyloidosis | E85.0 | Ever | 277.31 |
| Rapidly progressive nephritic syndrome | N01* | Ever | 580.4 |
| Syphilis of kidney and ureter | A52.75 | Ever | 095.4 |
| Tuberculosis of kidney and ureter | A18.11 | Ever | 016.00, 016.20 |
| *Plasmodium malariae* malaria with nephropathy | B52.0 | Ever | 084.9 with 581.81 |
| Recurrent and persistent hematuria | N02* | Ever | 581.1, 581.2, 581.3, 581.89, 581.9 |
| Chronic nephritic syndrome | N03* | Ever | 582.0, 582.1, 582.2, 582.4, 582.89, 582.9 |
| Nephrotic syndrome with diffuse mesangial proliferative glomerulonephritis | N04.3 | Ever | 581.2 |
| Nephrotic syndrome with diffuse endocapillary proliferative glomerulonephritis | N04.4 | Ever | 581.0, 581.2 |
| Nephrotic syndrome with diffuse mesangiocapillary glomerulonephritis | N04.5 | Ever | 581.2 |
| Nephrotic syndrome with dense deposit disease | N04.6 | Ever | 581.2 |
| Nephrotic syndrome with diffuse crescentic glomerulonephritis | N04.7 | Ever | 581.89 |
| Nephrotic syndrome with other morphologic changes | N04.8 | Ever | 581.89 |
| Nephrotic syndrome with unspecified morphologic changes | N04.9 | Ever | 581.9 |
| Unspecified nephritic syndrome | N05* | Ever | 583.1, 583.2, 583.89 |
| Hereditary nephropathy - not elsewhere classified | N07* | Ever | 583.1, 583.2, 583.89 |
| Glomerular disorders in diseases classified elsewhere | N08* | Ever | 583.81 |
| Chronic tubulo-interstitial nephritis | N11* | Ever | 590.00, 590.01, 590.80, 593.3, 593.4 |
| Analgesic nephropathy | N14.0 | Ever | 583.89 |
| Nephropathy induced by other drugs, medicaments and biological substances | N14.1* | Ever | 583.89 |
| Nephropathy induced by unspecified drug, medicament or biological substance | N14.2 | Ever | 583.89 |
| Nephropathy induced by heavy metals | N14.3 | Ever | 583.89 |
| Other renal tubulo-interstitial diseases | N15* | Ever | 583.89, 590.2 |
| Renal tubulo-interstitial disorders in systemic connective tissue disorders | N16* | Ever | 583.81, 590.81 |
| Chronic kidney disease | N18* | Ever | 585.1, 585.2, 585.3, 585.4, 585.5, 585.6 |
| Disorders resulting from impaired renal tubular function | N25.0, N25.1, N25.81, N25.89, N25.9 | Ever | 588.0, 588.1, 588.81, 588.89, 588.9 |
| Unspecified contracted kidney | N26* | Ever | 587, 405.91 |
| Cystic kidney disease | Q61.02, Q61.11, Q61.19, Q61.2, Q61.3, Q61.4, Q61.5, Q61.8 | Ever | 753.14, 753.13, 753.12, 753.15, 753.16, 753.17, 753.19 |
| Alport syndrome | Q87.81 | Ever | 759.89 |
| Hypertensive chronic kidney disease | I12* | Ever | 403.00, 403.01, 403.10, 403.11, 403.90, 403.91 |
| Hypertensive heart and chronic kidney disease | I13* | Ever | 404.00, 404.01, 404.02, 404.03, 404.10, 404.11, 404.12, 404.13, 404.90, 404.91, 404.92, 404.93 |
| Cloudy (hemodialysis) (peritoneal) dialysis effluent | R88.0 | Ever | 792.5 |
| Encounter for care involving renal dialysis | Z49* | Ever | V56.1, V56.2, V56.31, V56.32 |
| Dependence on renal dialysis | Z99.2 | Ever | V45.11 |
| Abnormal results of kidney function studies | R94.4 | Past 1 year | 794.4 |
| Sjögren syndrome with tubulo-interstitial nephropathy | M35.04 | Ever | 710.2 with 583.81 |
| Patient’s noncompliance with renal dialysis | Z91.15 | Past 5 years | V45.12 |
|  |  |  |  |
| **Diabetes** |  |  |  |
| Encounter for fitting and adjustment of insulin pump | Z46.81 | Ever | V53.91, V65.46 |
| Presence of insulin pump (external) (internal) | Z96.41 | Ever | V45.85 |
| Diabetes mellitus due to underlying condition | E08* | Past 5 years | 249.20, 249.10, 249.30, 249.40, 249.50, 249.90 |
| Drug or chemical induced diabetes mellitus | E09* | Past 5 years | 249.20, 249.10, 249.30, 249.40, 249.50, 249.60, 249.70, 249.80, 249.00 |
| Type 1 diabetes mellitus | E10* | Past 5 years | 250.11, 250.31, 250.41, 250.51 |
| Type 2 diabetes mellitus | E11* | Past 5 years | 250.00, 250.10, 250.20, 250.40, 250.50, 250.60, 250.70, 250.80, 250.90 |
| Other specified diabetes mellitus | E13* | Past 5 years | 249.10, 249.20, 249.30, 249.40, 249.50, 249.60, 249.70, 249.80, 249.90, 250.10, 250.20, 250.30, 250.40, 250.50, 250.60, 250.70, 250.80, 250.90 |
|  |  |  |  |
| **Immunocompromised status** |  |  |  |
| *Transplant* |  |  |  |
| Transplanted organ and tissue status | Z94* | Ever | V42.0, V42.1, V42.3, V42.4, V42.5, V42.6, V42.7, V42.81, V42.82, V42.83, V42.84, V42.89 |
| Complications of transplanted organs and tissue | T86* | Ever | 996.51, 996.52, 996.69, 996.79, 996.85, 996.81, 996.82, 996.83, 996.84, 996.86, 996.87, 996.88, 996.89 |
|  |  |  |  |
| *Malignancy* |  |  |  |
| Hodgkin lymphoma | C81* | Past 2 years | 201.00 – 201.08, 201.10 – 201.18, 201.20 – 201.28, 201.40 – 201.48, 201.50 – 201.58, 201.60 – 201.68, 201.70 – 201.78 |
| Follicular lymphoma | C82* | Past 2 years | 202.00 – 202.08, 202.80 – 202.88 |
| Non-follicular lymphoma | C83* | Past 2 years | 200.00 – 200.08, 200.10 –   200.18, 200.20 – 200.28, 200.30, 200.50 – 200.58, 200.70 – 200.78, 200.80 – 200.88 |
| Mature T/NK-cell lymphomas | C84* | Past 2 years | 200.60 – 200.68, 202.10 – 202.18, 202.20 – 202.28, 202.70 – 202.78, 202.80 – 202.88 |
| Other specified and unspecified types of non-Hodgkin lymphoma | C85* | Past 2 years | 200.70 – 200.78, 202.80 – 202.88, |
| Other specified types of T/NK-cell lymphoma | C86* | Past 2 years | 202.81, 202.87, 202.83, 202.80 |
| Malignant immunoproliferative diseases and certain other B-cell lymphomas | C88* | Past 2 years | 273.3, 203.80, 203.81, 200.30, 238.79 |
| Multiple myeloma and malignant plasma cell neoplasms | C90* | Past 2 years | 203.00 – 203.02, 203.10 – 203.12, 203.80 – 203.82 |
| Lymphoid leukemia | C91* | Past 2 years | 204.00 – 204.02, 204.10 – 204.12, 204.80 – 204.82, 202.40, 204.90 – 204.92 |
| Myeloid leukemia | C92* | Past 2 years | 205.00 – 205.02, 205.10 – 205.12, 205.20 – 205.22, 205.30 – 205.32, 205.80 – 205.82, 205.90 – 205.92 |
| Monocytic leukemia | C93* | Past 2 years | 206.00 – 206.02, 206.10 – 206.12, 206.20 – 206.22, 206.80 – 206.82, 206.90 – 206.92 |
| Other leukemias of specified cell type | C94* | Past 2 years | 207.00 – 207.02, 207.20 – 207.22, 207.80 – 207.82, 238.79 |
| Leukemia of unspecified cell type | C95* | Past 2 years | 208.00 – 208.02, 208.10 – 208.12, 208.20 – 208.22, 208.80 – 208.82, 208.90 – 208.92 |
| Other and unspecified malignant neoplasms of lymphoid, hematopoietic and related tissue | C96* | Past 2 years | 202.30, 202.50, 202.60, 202.90 – 202.92, 277.89 |
|  |  |  |  |
| *Immunosuppressive medications* |  |  |  |
| Personal history of immunosuppression therapy | Z92.25 | Past 6 months | V87.46 |
| Encounter for antineoplastic radiation therapy | Z51.0 | Past 6 months | V58.0 |
| Encounter for antineoplastic chemotherapy and immunotherapy | Z51.1 | Past 6 months | V58.11, V58.12 |
|  |  |  |  |
| *Other immunodeficiencies* |  |  |  |
| Functional disorders of polymorphonuclear neutrophils | D71* | Past 10 years | 288.1 |
| Immunodeficiency with predominantly antibody defects | D80* | Past 10 years | 279.00, 279.01, 279.02, 279.03, 279.04, 279.05, 279.09 |
| Combined immunodeficiencies | D81* | Past 10 years | 279.13, 279.2 |
| Immunodeficiency associated with other major defects | D82* | Past 10 years | 279.11, 279.12, 279.8 |
| Common variable immunodeficiency | D83* | Past 10 years | 279.06 |
| Other immunodeficiencies | D84* | Past 10 years | 277.6, 279.8, 279.10, 279.19, 279.3 |

^a^ Definition of high-risk conditions were not mutually exclusive. For example, an individual with chronic obstructive pulmonary disease could also have had heart failure. In such a case, that individual would have been counted in both the “chronic obstructive pulmonary disease” category and the “heart failure” category.

^b^ Time frame looked backwards in time beginning with the index date, which September 1 immediately preceding the start of that winter respiratory virus season. For example, for a condition that must be observed within the past 5 years, a participant in the vaccine effectiveness study during the 2018-19 season must have at least 1 ICD code observed within the 5 years preceding September 1. 2018.

*Asterisks indicate the inclusion of all constituent (“child”) codes underneath the parent code with the asterisk.
