# Supplementary material for: Seasonal Incidence of Human Metapneumovirus in High‐Risk Adults With Medically Attended Acute Respiratory Illness in a Rural US Community
Source: Influenza Other Respir Viruses. 2025 Jul 17;19(7):e70119. doi: 10.1111/irv.70119 (PMC12268109; doi:10.1111/irv.70119)
Supplement: Supplementary file 3 — Table S3. ICD codes used for definitions of specific high‐risk subcategories among individuals in the high‐risk adult cohort. [file IRV-19-e70119-s004.docx]

**Supplemental Table 3**. ICD codes used for definitions of specific high-risk subcategories among individuals in the high-risk adult cohort.

| Sub-categorization | ICD-10 codes used | ICD-9 codes used |
| --- | --- | --- |
| Cardiac disorders - Congestive heart failure | I09.81, I11.0, I13.0, I13.2,^a^ I50, I50.1, I50.2, I50.20, I50.21, I50.22, I50.23, I50.3, I50.30, I50.31, I50.32, I50.33, I50.4, I50.40, I50.41, I50.42, I50.43, I50.8, I50.81, I50.810, I50.811, I50.812, I50.813, I50.814, I50.82, I50.83, I50.84, I50.89, I50.9 | 398.91, 402.01, 402.11, 402.91, 404.01, 404.11, 404.91, 404.03, 404.13, 404.93, 428.0, 428.1, 428.20, 428.21, 428.22, 428.23, 428.30, 428.31, 428.32, 428.33, 428.40, 428.41, 428.42, 428.43, 428.9 |
| Cardiac disorders - Coronary artery disease including myocardial infarction | I20, I20.0, I20.1, I20.8, I20.9, I21, I21.0, I21.01, I21.02, I21.09, I21.1, I21.11, I21.19, I21.2, I21.21, I21.29, I21.3, I21.4, I21.9, I21.A, I21.A1, I21.A9, I22, I22.0, I22.1, I22.2, I22.8, I22.9, I23.0, I23.1, I23.2, I23.3, I23.4, I23.5, I23.6, I23.7, I23.8, I24, I24.0, I24.1, I24.8, I24.9, I25.1, I25.10, I25.11, I25.110, I25.111, I25.118, I25.119, I25.2, I25.3, I25.41, I25.42, I25.5, I25.6, I25.700, I25.701, I25.708, I25.709, I25.710, I25.711, I25.718, I25.719, I25.720, I25.721, I25.728, I25.729, I25.730, I25.731, I25.738, I25.739, I25.75, I25.750, I25.751, I25.758, I25.759, I25.760, I25.761, I25.768, I25.769, I25.790, I25.791, I25.798, I25.799, I25.810, I25.811, I25.812, I25.82, I25.83, I25.84, I25.89, I25.9, Z95.1, Z95.5, Z98.61 | 413.0, 413.1, 413.9, 410.*, 429.79, 429.71, 429.5, 429.6, 411.81, 411.0, 411.89, 414.01, 414.02, 414.03, 414.04, 414.05, 414.06, 414.07, 414.10, 414.11, 414.12, 414.19, 414.2, 414.3, 414.4, 414.8, 414.9, 429.2, 412, 411.1, V45.81, V45.82 |
| Cardiac disorders - Arrhythmias | I44, I44.0, I44.1, I44.2, I44.3, I44.30, I44.39, I44.4, I44.5, I44.6, I44.60, I44.69, I44.7, I45, I45.0, I45.1, I45.10, I45.19, I45.2, I45.3, I45.4, I45.5, I45.6, I45.8, I45.81, I45.89, I45.9, I48, I48.0, I48.1, I48.2, I48.3, I48.4, I48.9, I48.91, I48.92, I49.1, I49.2, I49.3, I49.4, I49.40, I49.49, I49.5, I49.8, I49.9,  R00.0, R00.1, R00.2, Z45.0, Z45.01, Z45.010, Z45.018, Z45.02, Z45.09, Z95.0, Z95.810 | 426.11, 426.12, 426.13, 426.0, 426.10, 426.50, 426.2, 426.3, 426.4, 426.50, 426.51, 426.52, 426.53, 426.54, 426.6, 426.7, 426.81, 426.82, 426.89, 426.9, 427.31, 427.32, 427.41, 427.42, 427.61, 427.0, 427.69, 427.60, 427.69, 427.81, 427.89, 427.9, 785.0, 427.81, 427.89, 785.1, V45.01, V45.02, V53.31, V53.32, V53.39 |
| Respiratory disorders - COPD | J41, J41.0, J41.1, J41.8, J42, J43, J43.0, J43.1, J43.2, J43.8, J43.9, J44, J44.0, J44.1, J44.9, J47, J47.0, J47.1, J47.9 | 491.0, 491.1, 491.8  , 491.9, 492.0, 492.8, 491.22, 493.21, 491.21, 493.22, 491.20, 493.20, 496, 494.0, 494.1 |
| Respiratory disorders - Asthma | J45, J45.2, J45.20, J45.21, J45.22, J45.3, J45.30, J45.31, J45.32, J45.4, J45.40, J45.41, J45.42, J45.5, J45.50, J45.51, J45.52, J45.9, J45.90, J45.901, J45.902, J45.909, J45.99, J45.990, J45.991, J45.998 | 493.00, 493.01, 493.02, 493.10, 493.11, 493.12, 493.81, 493.82, 493.90, 493.91, 493.92 |
| Respiratory disorders - Cystic fibrosis | E84.0, E84.11, E84.19, E84.8, E84.9 | 277.01, 277.02, 277.03, 277.00, 277.09 |

^a^ I13.0 and I13.2 correspond to codes relating to hypertensive heart and chronic kidney disease and heart failure. These codes are therefore used both to identify someone as having heart failure and as having chronic kidney disease.
