# Supplementary material for: Seasonal Incidence of Human Metapneumovirus in High‐Risk Adults With Medically Attended Acute Respiratory Illness in a Rural US Community
Source: Influenza Other Respir Viruses. 2025 Jul 17;19(7):e70119. doi: 10.1111/irv.70119 (PMC12268109; doi:10.1111/irv.70119)
Supplement: Supplementary file 4 — Table S4. Summary statistics for population‐weighting factors in the high‐risk adult cohort and high‐risk adult source population. [file IRV-19-e70119-s003.docx]

**Supplemental Table 4.** Summary statistics for population-weighting factors in the high-risk adult cohort and high-risk adult source population.

|  | High-risk adult cohort  N = 3,601 | High-risk adult source population  N = 251,413 |
| --- | --- | --- |
| Winter respiratory virus season [adjustment factor]^a^ : n (%) |  |  |
| 2015-16 [1.35] | 491 (13.6) | 42,523 (16.9) |
| 2016-17 [1.10] | 695 (19.3) | 41,960 (16.7) |
| 2017-18 [1.43] | 867 (24.1) | 41,574 (16.5) |
| 2018-19 [1.52] | 722 (20.1) | 62,941 (25.0) |
| 2019-20 [1.20] | 826 (22.9) | 62,415 (24.8) |
| Age group: n (%) |  |  |
| 18-49 years | 1,457 (40.5) | 72,956 (29.0) |
| 50-59 years | 697 (19.4) | 44,390 (17.7) |
| 60-74 years | 926 (25.7) | 77,576 (30.9) |
| ≥75 years | 521 (14.5) | 56,491 (22.5) |
| Female sex: n (%) | 2,358 (65.5) | 139,960 (55.7) |
| Residence within MESA Central^b^: n (%) | 2,596 (72.1) | 100,833 (40.1) |
| Number of medically-attended acute respiratory illness visits during the respiratory virus season: median (IQR) | 1.0 (1.0 – 2.0) | 0.0 (0.0 – 0.0) |
| Number of MAARI visits during the winter respiratory virus season: n (%) |  |  |
| 0^d^ | 277 (7.7) | 204,377 (81.3) |
| 1 | 1,744 (48.4) | 28,293 (11.2) |
| ≥2 | 1,580 (43.9) | 18,743 (7.5) |

^a^ Adjustment factor presented is a factor to adjust for estimated hMPV season length (versus portion of the season believed to have been captured by the Flu VE study), based on Wisconsin State Laboratory of Hygiene (WSLH) reports of hMPV circulation by year. The WSLH-based estimated season lengths are not specific to adults with high-risk conditions.

^b^ Marshfield Epidemiologic Study Area Central, a geographic area covering approximately 60,000 residents who live in the 14 ZIP codes surrounding Marshfield, Wisconsin.

^c^ A MAARI visit of 0 means that an individual had no medically-attended acute respiratory illness visits in a given winter respiratory virus season. Among Flu VE study enrollees, this was a rare circumstance that may have occurred when certain individuals were approached to be enrolled the day after a clinic visit where they had a respiratory illness, but did not receive a MAARI billing code.
